# Morpho-Molecular Metabolic Analysis and Classification of Human Pituitary Gland and Adenoma Biopsies Based on Multimodal Optical Imaging

**DOI:** 10.3390/cancers13133234

**Published:** 2021-06-29

**Authors:** Gabriel Giardina, Alexander Micko, Daniela Bovenkamp, Arno Krause, Fabian Placzek, Laszlo Papp, Denis Krajnc, Clemens P. Spielvogel, Michael Winklehner, Romana Höftberger, Greisa Vila, Marco Andreana, Rainer Leitgeb, Wolfgang Drexler, Stefan Wolfsberger, Angelika Unterhuber

**Affiliations:** 1 Center for Medical Physics and Biomedical Engineering, Medical University of Vienna, Waehringer Guertel 18-20, 1090 Vienna, Austria; gabriel.giardina@meduniwien.ac.at (G.G.); daniela.bovenkamp@meduniwien.ac.at (D.B.); arno.krause@meduniwien.ac.at (A.K.); fabian.placzek@meduniwien.ac.at (F.P.); rainer.leitgeb@meduniwien.ac.at (R.L.); wolfgang.drexler@meduniwien.ac.at (W.D.); angelika.unterhuber@meduniwien.ac.at (A.U.); 2 Department of Neurosurgery, Medical University of Vienna, Waehringer Guertel 18-20, 1090 Vienna, Austria; alexander.micko@meduniwien.ac.at (A.M.); stefan.wolfsberger@meduniwien.ac.at (S.W.); 3 QIMP Team, Center for Medical Physics and Biomedical Engineering, Medical University of Vienna, Waehringer Guertel 18-20, 1090 Vienna, Austria; laszlo.papp@meduniwien.ac.at (L.P.); denis.krajnc@meduniwien.ac.at (D.K.); 4 Christian Doppler Laboratory for Applied Metabolomics, Division of Nuclear Medicine, Medical University of Vienna, Waehringer Guertel 18-20, 1090 Vienna, Austria; clemens.spielvogel@meduniwien.ac.at; 5 Division of Neuropathology and Neurochemistry, Department of Neurology, Medical University of Vienna, Waehringer Guertel 18-20, 1090 Vienna, Austria; michael.winklehner@meduniwien.ac.at (M.W.); romana.hoeftberger@meduniwien.ac.at (R.H.); 6 Department of Internal Medicine III, Division of Endocrinology and Metabolism, Medical University of Vienna, Waehringer Guertel 18-20, 1090 Vienna, Austria; greisa.vila@meduniwien.ac.at

**Keywords:** pituitary gland and adenomas, multimodal imaging, Raman spectroscopy, second harmonic generation, two-photon excitation fluorescence, multiphoton microscopy, optical coherence tomography, image analysis, radiomics

## Abstract

**Simple Summary:**

The pituitary gland governs the function of nearly all endocrine glands and pituitary oncogenesis often distorts the hormonal balance. For optimal surgical cure it is crucial to discriminate pathological tissue from intact pituitary gland. Our multimodal imaging approach allows for morpho-molecular metabolic analysis and discrimination of pituitary gland and adenomas combining different complementary techniques such as optical coherence tomography (OCT), multiphoton microscopy (MPM) and line scan Raman microspectroscopy (LSRM). Radiomics as well as analysis of spectroscopic features allows enhanced discrimination of pituitary gland and adenomas and, furthermore, classification of pituitary adenoma subtypes.

**Abstract:**

Pituitary adenomas count among the most common intracranial tumors. During pituitary oncogenesis structural, textural, metabolic and molecular changes occur which can be revealed with our integrated ultrahigh-resolution multimodal imaging approach including optical coherence tomography (OCT), multiphoton microscopy (MPM) and line scan Raman microspectroscopy (LSRM) on an unprecedented cellular level in a label-free manner. We investigated 5 pituitary gland and 25 adenoma biopsies, including lactotroph, null cell, gonadotroph, somatotroph and mammosomatotroph as well as corticotroph. First-level binary classification for discrimination of pituitary gland and adenomas was performed by feature extraction via radiomic analysis on OCT and MPM images and achieved an accuracy of 88%. Second-level multi-class classification was performed based on molecular analysis of the specimen via LSRM to discriminate pituitary adenomas subtypes with accuracies of up to 99%. Chemical compounds such as lipids, proteins, collagen, DNA and carotenoids and their relation could be identified as relevant biomarkers, and their spatial distribution visualized to provide deeper insight into the chemical properties of pituitary adenomas. Thereby, the aim of the current work was to assess a unique label-free and non-invasive multimodal optical imaging platform for pituitary tissue imaging and to perform a multiparametric morpho-molecular metabolic analysis and classification.

## 1. Introduction

Pituitary adenomas account for 15% of all intracranial tumors [1,2,3,4] and are associated with significant morbidity and increased mortality by virtue of hormone hyper- and hyposecretion, even though they show a benign behavior in the majority of cases [5]. The incidence rate of pituitary adenomas in the general population is reported to be approximately 4 per 100,000 [6]. According to the meta-analysis of Ezzat et al. [2], prolactin (PRL) cell adenomas account for 25–41% of all cases, followed by non-functioning adenomas (35%), growth hormone (GH) cell adenomas (10–15%), adrenocorticotropic hormone (ACTH) cell adenomas (15%) and thyroid stimulating hormone (TSH) cell adenomas (2%). Population-based studies revealed that the peak age of pituitary adenomas is between 40 and 60 years and that the female-to-male ratio differs greatly between subtypes [7,8].

The pituitary gland is situated within the skull base, underneath the brain and in connection with the hypothalamus and represents the primary control unit of hormonal production of the body. Due to various feedback mechanisms, the pituitary gland regulates several other endocrine glands within the body, thereby playing a crucial role in growth, metabolism and reproductive function. This role mandates an accurate diagnostic for safe and effective disease treatment. Decision-making of treatment is critically dependent on the imaging quality. Magnetic resonance imaging is the standard radiologic procedure used for preoperative diagnosis, but its intraoperative use is time consuming, cost-intensive and delivers a high incidence of false-positive results. The goal of modern pituitary surgery is to remove the adenoma entirely while preserving normal gland function. In this context, an overly cautious resection carries the risk of tumor remnants and associated recurrence [9]. In contrast, a too aggressive surgical resection may lead to damage of the pituitary gland and thus may be associated with a lifelong substitution therapy. Tissue diagnostics in clinical routine settings is performed by neuropathologists via microscopic analysis of the processed, sectioned and stained surgically removed tissue biopsy. Histological processing is cost-, labor- and by that as well time-intensive. Furthermore, the histological treatment of tissue is often associated with distortion artifacts and reduced in vivo biological information. Hence, accurate three-dimensional (3D) image reconstruction and real-time intra-surgical tissue investigation is often not possible.

Despite recent advancements in histopathological procedures, there is an increasing demand for efficient, accurate and less complex diagnostic processes that would allow for direct intra-surgical assessment. Such processes can potentially help determine the size, dimensions and margins of the tumor more accurately. To support this endeavor, novel imaging approaches for intraoperative assessment of the extend of resection are required. Powerful label-free spectroscopic and microscopic optical imaging techniques such as Raman spectroscopy (RS) [10], multiphoton microscopy (MPM) [11] and optical coherence tomography (OCT) [12] have been developed and widely used in biomedical applications over the past years. These modalities offer similar contrast compared to standard histopathological methods [13]. Recently, there has been an increasing interest in applying and especially fusing these complementary techniques in medical diagnostics to investigate a wide range of cancers to obtain qualitative and quantitative 3D morphological, metabolic and molecular information without the need for sample preparation, in a non-invasive and label-free way.

RS can provide an intrinsic molecular fingerprint of the tissue and therefore offers the potential to capture early molecular changes. MPM is the umbrella term for two-photon excitation fluorescence (TPEF) and second harmonic generation (SHG) microscopy providing endogenous contrasts of cancer tissue with intrinsic 3D sectioning and increased field of view (FOV) compared to RS. TPEF signals arise from a variety of endogenous fluorescent molecules in cells and in the extracellular matrix, predominately nicotinamide adenine dinucleotide (NADH) and flavin adenine dinucleotide (FAD) [14] and as a result it is able to provide metabolic information. SHG can be easily implemented on the same platform with intrinsic co-registration to obtain specific morphological tissue information. SHG signals arise from non-centrosymmetric molecules in tissue such as collagen fibers [15]. OCT exceeds RS and MPM in terms of speed, FOV and penetration depth. It is sensitive to changes in the refractive index and is thereby an ideal candidate to visualize textural patterns and structural features [16].

In previous studies, the pituitary gland has been analyzed with single optical modalities demonstrating promising results but with some limitations. Lin et al. demonstrated MPM on the pituitary gland, hyperplasia and pituitary adenomas [17,18]. SHG and TPEF displayed the different distribution patterns of pituitary, tumor and blood cells and the morphological changes and amount of reticular fibers with SHG. The consistency of pituitary adenomas could be predicted and the ratio between SHG and TPEF signals, as well the collagen content alone, could be used as quantitative biomarkers. However, these studies could not be performed on thick tissue and required thin slicing. Moreover, as MPM cannot decipher the full molecular fingerprint, it lacks hormone classification as it is used in clinical practice. Additionally, the screening of large areas or even volumes remains a challenge. Placzek et al. demonstrated the potential of OCT on pituitary gland and pituitary adenomas [19]. Morphological biomarkers for pituitary gland and pituitary adenoma differentiation could be established but metabolic and molecular information were lacking. OCT was correlated with H&E staining and collagen was identified as main biomarker. LSRM has been shown to give promising results in differentiation of pituitary gland, different pituitary adenomas as well as discrimination to the surrounding tissue, the periosteal layer [20]. Spectral analysis was able to discriminate between the subclasses of pituitary adenomas. However, as this technique is intrinsically slow, large area and depth resolved 3D screening is not possible with this technique.

No single mentioned optical imaging modality is capable to obtain all information provided by histological and immunohistochemical analysis and each modality has its own limitations. RS is very slow and acquisition times per point are in the order of ~ 1 s or longer resulting in imaging speeds in the order of hours. MPM requires the acquisition of multiple images to obtain metabolic information. OCT provides real-time 3D morphological imaging up to several millimeters in depth and cm^2^ in terms of FOV but does not provide information about the metabolic state or molecular distribution. In combination, all modalities together have the potential to go beyond the limitation of each optical imaging method alone and thus provide ultrahigh resolution 3D structural, metabolic and molecular information in a label-free manner without any tissue distortion and at the same time.

The interpretation of such mosaic of information provided by different optical imaging modalities is not trivial. Indeed, in recent years, radiomics has demonstrated to be a useful approach to characterize imaging pattern characteristics in vivo [21]. Radiomics refers to the process of extracting various numerical features from medical images to identify potential imaging biomarkers among them that are descriptive to the given disease. Given that most radiomic features characterize tumor heterogeneity, it is a prime candidate approach to analyze tumors in various medical images [22,23].

In the present work, we aim to show the potential of a unique multimodal optical imaging platform combining OCT, MPM and LSRM for pituitary tissue imaging with the capability of providing conclusive morphological, metabolic and molecular 3D tissue information on a cellular level. The combination of these unique tissue features are objectively performed by radiomic analysis. Moreover, we evaluate the possibility for enhanced discrimination of pituitary gland and pituitary adenoma subtypes from biopsied tissue from a cohort of patients with a multiparametric biomarker and radiomics combination.

## 2. Materials and Methods

### 2.1. Pituitary Specimen and Ethics

Pituitary adenomas, including lactotroph (*n* = 5), null cell (*n* = 5), gonadotroph (*n* = 5), somatotroph and mammosomatotroph (*n* = 5) as well as corticotroph (*n* = 5), and adjacent pituitary gland tissue (*n* = 5) were excised during purely endoscopic transsphenoidal surgery by the Department of Neurosurgery of the Medical University of Vienna (MUV). Due to their poor prevalence, thyrotrophic adenomas were not included in this study. This study as well as the procedures were approved by the ethics committee of the MUV (code: EK1286/2018). Prior to enrollment in the study, written informed consent was collected from all participants.

The multimodal measurements of the pituitary gland and adenomas were performed at room temperature on thawed cryogenic samples (−80 °C) which were frozen immediately after surgery to ensure conditions comparable to fresh biopsies. The specimens’ sizes ranged from 1 mm to 2 mm in diameter for pituitary gland samples and 1 mm to 3 mm for adenoma tissue. After the measurements, the tissues were immediately fixed in neutral buffered formalin and transferred to the Division of Neuropathology and Neurochemistry for further histopathological analysis by neuropathologists. Hematoxylin and eosin (H&E) and Elastica van Gieson (EVG) staining was performed. The histopathological diagnosis was available for each biopsy. Histopathological analysis was performed according to the criteria of the latest WHO classification of pituitary adenomas [24] using immunohistochemical staining for hormones as well as transcription factors [25].

### 2.2. Multimodal OCT, MPM and LSRM System

**Figure 1:**
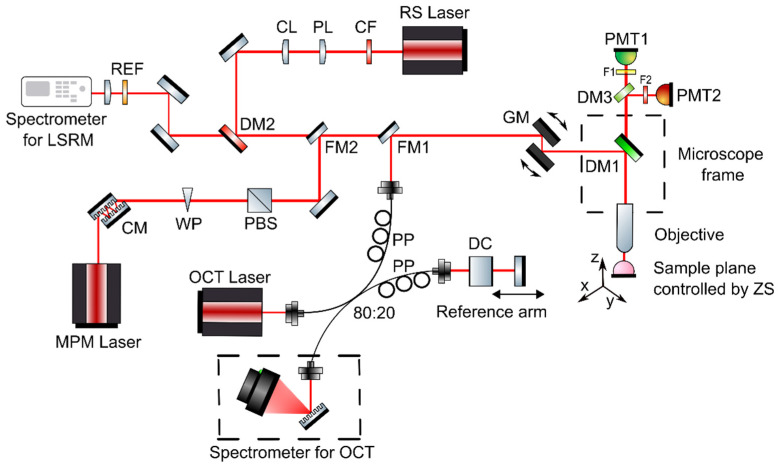
Multimodal optical system featuring OCT, MPM as well as LSRM. The three modalities are coupled into the microscope frame via one common optical path with flipping mirrors. Abbreviations: CF—clean up filter, PL—Powell lens, CL—cylindrical lens, DM—dichroic mirrors, REF—razor edge filter, CM—chirp mirrors, WP—half-wave plate, PBS—polarization beam splitter, DC—dispersion compensating glass, PP—polarization paddles, FM—flipping mirror, GM—galvanometric mirrors, F—filters, PMT—photomultiplier tube, ZS—Zaber stage.

The developed multimodal optical imaging platform featuring ultrahigh-resolution spectral domain (UHR-SD) OCT, MPM including contrast from SHG and TPEF, and LSRM was built into a customized upright laser scanning microscope (Eclipse E400, Nikon, Tokyo, Japan). Galvanometric mirrors were implemented for scanning the sample plane (6230H, Cambridge Technology, Bedford, MA, USA) and navigating through the sample. A 3D motorized translation stage (X-LSM050A-E, Zaber, Vancouver, BC, Canada) was used to additionally move the specimen. A dichroic mirror directed the excitation beams through the objective onto the tissue sample and separated MPM excitation and collection. For all three modalities, OCT, MPM and LSRM, a common optical path and the same microscope objective was used (16×, CFI LWD Plan Fluorite Objective, 0.8 numerical aperture (NA), Nikon, Japan) to facilitate navigation on the sample and co-registration. Furthermore, the high NA objective was chosen to provide ultrahigh almost isotropic resolution and efficient collection of MPM and Raman signals. The combination of a customized MATLAB software and ScanImage [26] made it possible to control the galvanometric mirrors, the motorized stages, to set and position the laser beams and biopsies as well as to start excitation and acquisition. Via flipping mirrors, each modality was coupled into the common optical path in front of the galvanometric mirrors of the microscope frame as illustrated in Figure 1. As each technique makes it possible to assess different complementary tissue information, Table 1 gives an overview of the excitation wavelengths, the detection, lateral, axial and spectral resolution and complementary contrast mechanism.

#### 2.2.1. Optical Coherence Tomography Arm

The UHR-SD OCT system implemented in the microscope frame has been described elsewhere and was modified to meet the requirements of this study [27,28]. In brief, a broadband Ti:Sapphire laser centered at 800 nm was used as light source for the UHR-SD OCT system. The power on the sample plane was set to 2.5 mW. For OCT signal detection, a spectrometer consisting of a 1200 l/mm grating (Wasatch Photonics, Logan, UT, USA) and 840 nm central wavelength, and a 12-bit CCD-based line scan camera with a maximum line rate of 70 kHz and 2048 pixels (AViiVA Atmel EM4CL 2014, Essex, UK) was used. The sensitivity of this system was measured to be 97 dB. Axial and lateral resolution were 2.2 µm and 1 µm in tissue, respectively. Areas of 562 × 562 µm^2^ were covered by the FOV. The pixel size in depth after FFT was measured to be 1.3 µm, resulting in a penetration of approximately 1 mm. Scanning was performed with galvanometric mirrors.

Acquisition and analysis software for OCT data was written in-house in MATLAB. OCT data was averaged 5 times. OCT software allowed for live preview which was running at 20 B-scans per second. Thereby, real-time processing allowed 3D navigation and screening across the sample without noticeable time lag.

#### 2.2.2. Multiphoton Microscopy Arm

The MPM light source featuring simultaneous SHG and TPEF signal generation was a customized tunable femtosecond Ti:Sapphire laser ranging from 750 nm to 950 nm. Optics in the MPM arm included dispersion pre-compensation with a pair of chirped mirrors to guarantee sub 100 fs pulses at the sample plane. Laser power was controlled with a combination of a half-wave plate and polarization beam splitter. A dichroic mirror (T495LPXR, Chroma Technology, Burlington, VT, USA) was used to separate the backward MPM signal from the excitation laser. MPM signals were detected in two different channels with photomultiplier tubes (H10723-01, Hamamatsu Photonics K. K., Japan). Two different bandpass filters (445/45 BrightLine HC, F37-446, Semrock Rochester, NY, USA and 550/49 BrightLine HC, F37-551, Semrock Rochester, NY, USA) were used to collect signals around 445 nm and 550 nm, respectively. Filters were chosen to spectrally discriminate signals from collagen and NADH in the 445 nm channel and FAD in the 550 nm channel. Furthermore, scanning was performed with the galvanometric mirrors on the same ROI than OCT. Two-dimensional acquisition rate was 1.2 frames/s covering a FOV of 508 × 508 µm^2^. The slightly different FOV is due to the not fully uniform illumination. Imaging was performed at two different wavelengths, 760 nm and 865 nm, respectively. NADH signals were observed at 445 nm upon 760 nm illumination and SHG signals at 430 nm upon 865 nm illumination. FAD signals were present at 550 nm upon 865 nm excitation. The dual wavelength detection allowed for calculation of the ratio between NADH and FAD signals [29,30]. Acquisition of MPM data was controlled via the free software ScanImage [26]. Analysis and further postprocessing was done with in-house written MATLAB scripts.

#### 2.2.3. Line Scan Raman Microspectroscopy Arm

For excitation, a customized continuous wave Ti:Sapphire laser was used. The laser power on the sample plane was 150 mW over the full illumination line. To form a uniform line illumination, a Powell lens and cylindrical lens (both Thorlabs, Newton, NJ, USA) were implemented to shape the laser beam. The parameters of the spectrometer (Shamrock SR 303i, Andor Technology, Belfast, Northern Ireland) are described elsewhere [20]. In brief, the 300 l/mm grating blazed at 500 nm in the spectrometer had a spectral resolution of 0.5 nm. The CCD-camera with 255 × 1024 pixels (Newton 920i, Andor Technology, Belfast, Northern Ireland) was used for detection of LSRM spectra. The spectrometer had the slit size set to 100 µm. Filters in the LSRM system included a laser clean-up filter (785 nm MaxLine^®^, LL01-785-12.5, Semrock, Rochester, NY, USA) and an ultra-steep long-pass edge filter (785 nm RazorEdge^®^, LP02-785RE-25, Semrock, Rochester, NY, USA). A long-pass dichroic mirror with a cut-on wavelength of 805 nm (DMLP805, Thorlabs, Newton, NJ, USA) separated the Raman signal from the excitation laser. The biopsies were moved with a linear stage (X-LSM050A-E, Zaber, Vancouver, Canada) with a step size of 5 µm to create a 2D image. Raman spectra were simultaneously collected along the laser line covering an area of 80 µm × 250 µm in 10 min.

The analysis procedure for raw Raman spectra followed the protocol established by Bocklitz et al. [31]. Briefly, fluorescent background was removed by fitting an iterative polynomial algorithm [32], followed by smoothing using Savitzky-Golay filtering [33] and 0–1 normalization to the C-H band at 2945 cm^−1^. Discrimination of Raman spectra and training of classifier algorithms was performed by principal component analysis (PCA) followed by a support vector machine (SVM) in MATLAB [34,35].

#### 2.2.4. Statistical and Radiomic Analysis

We performed statistical analysis per optical modality between pituitary gland and each adenoma subtype (*n* = 5). Shapiro-Wilk W test for normality distribution was initially performed accordingly to Royston et al. [36]. The null hypothesis was not rejected at a significance level of 0.05 in all cases. Furthermore, two-tailed Welch’s *t*-test was used, and thereby *p*-values smaller than 0.05 were considered as significantly different.

Statistical test for evaluation of the significance of Raman ratios were performed by analysis of variance (ANOVA), thereby *p*-values smaller than 0.05 were considered as significantly different.

OCT, TPEF, SHG and MPM (combination of TPEF and SHG information) images with a size of 128 × 128 pixels were subject of radiomic analysis. Each image of all collected samples underwent a region of interest mask generation upon visual inspection removing void background. The respective masks of OCT, TPEF, SHG and MPM were used to determine which pixels of each modality should be part of the analysis. Binning of the above images was performed with a cohort-global bin width per modality. Here, the largest bin width was selected per modality which did not result in completely homogeneous (number of bins 1) regions in the cohort (bin widths OCT: 2.78, TPEF: 1.25, MPM: 1.3, SHG: 1.28). The MUW Radiomics Engine ver. 2.0 [37] was used to extract a total number of 234 radiomic features from each sample as of optimized radiomic principles [38]. The resulting radiomic dataset underwent redundancy reduction with covariance matrix analysis where a Pearson correlation of 0.85 was selected as threshold [37]. This step resulted in 98 features.

A 100-fold balanced Monte Carlo (MC) cross-validation was used to generate training and validation subsets. For selecting validation samples in each fold, 20% of the minority subgroup size was calculated (N_Minority_) and N_Minority_ amount from each disease subtype was assigned to the validation dataset. The remaining data was assigned to the training dataset. Each MC fold was unique without allowing subset configuration repetitions. In each fold, feature ranking and selection of the highest-ranking 10 features was performed via R-squared ranking in the training set of each MC fold. The selected features were then also selected for the validation subset [37,39]. This step was necessary to minimize the chances of overfitting. The training set of each MC fold was further processed by Tomek Links [40] to remove borderline cases from the majority (adenomas) subtype. Last, the minority subtype (gland) of each training set underwent synthetic minority oversampling technique (SMOTE) to handle class imbalance before machine learning [41].

Each MC fold training set was the input of a mixed random forest ensemble classifier training process [37]. Once the radiomic features model was built, the validation set of the given MC fold was also evaluated to estimate predictive performance of the model on an independent dataset. Predictive performance was estimated via confusion matrix analytics across the MC folds.

## 3. Results

The results are organized in subsections showing three representative tissue samples of pituitary gland and two adenoma subtypes, mammosomatroph and gonadotroph adenomas, respectively. All multimodal images are represented in the same way and show the correlation of the morphological, metabolic and molecular information arising from co-registered OCT and MPM images as well as the corresponding LSRM images and mean spectra acquired with the multimodal platform. OCT orthogonal views show a representative OCT en face slide with corresponding cross-sections (B-scans). The blue dashed rectangle in the OCT en face slide indicates the region of interest where the LSRM scan was performed. In addition, white dotted rectangles in OCT B-scans indicate the positions of magnified regions of interest in B-scans. Green dashed lines (1 and 2) in these representations indicate the positions of overlaid OCT-MPM 2D image planes for radiomic analysis at two different depths. Collagen structures detected in the SHG channel are color-coded in pink. Metabolic information by means of NADH and FAD fluorescence was detected with TPEF and is color-coded in blue. Color-coding of SHG and TPEF signals was chosen based on the colors which are familiar in H&E-stained slices to provide a “virtual histology” [42]. Representative H&E-stained and corresponding EVG-stained images with contrast from connective tissue in pink are correlated with the fused multimodal images. The position of the LSRM FOV is marked with a dashed blue rectangle in the OCT en face images. LSRM images visualize the spatial distribution of various components, in particular collagen, proteins and lipids. The corresponding Raman bands are shown and marked with black dashed lines on the mean Raman spectra for each case.

**Figure 2:**
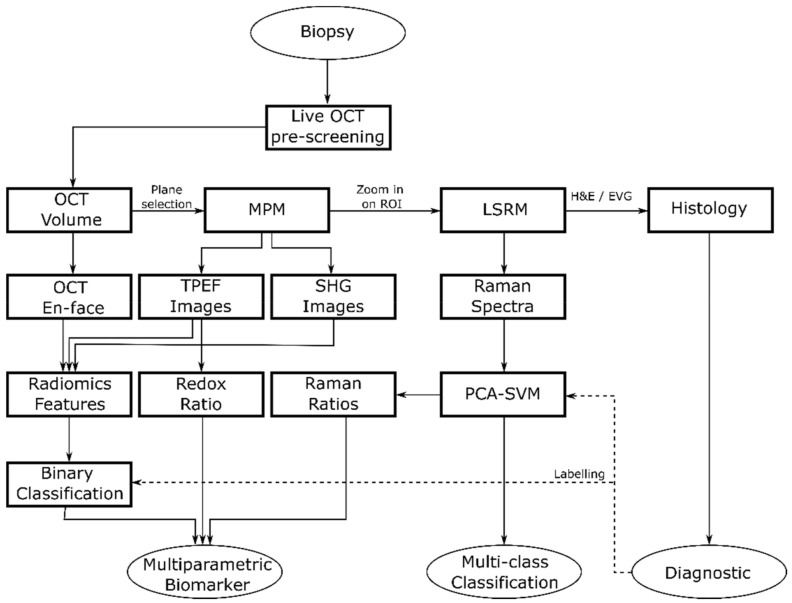
Flow diagram showing the general flow of the multimodal tissue measurement and data analysis.

We performed sequential imaging following a specific rationale illustrated in Figure 2. First, OCT was performed to provide a real time 3D navigational tool to identify suspicious regions within the biopsy down to ~300 µm in depth and to identify ROIs revealing discrimination features between pituitary gland and adenomas for fast preliminary volumetric binary classification. Next, MPM images were collected to identify initial molecular signatures. Finally, we performed LSRM to obtain additional information on the full molecular content from specific areas of interest allowing for further discrimination of pituitary adenoma subtypes. In particular, we scanned the laser line within the OCT-MPM overlay to provide an image from about 2100 collected spectra of the molecular fingerprint in a reasonable amount of time, instead of performing very long Raman mapping [43]. Since pathologists usually perform H&E staining as the gold standard in clinical practice as a first step in pituitary adenoma diagnostics, 2-µm-thick tissue sections of the biopsies were H&E-stained after the multimodal investigation to facilitate interpretation.

### 3.1. Pituitary Gland

**Figure 3:**
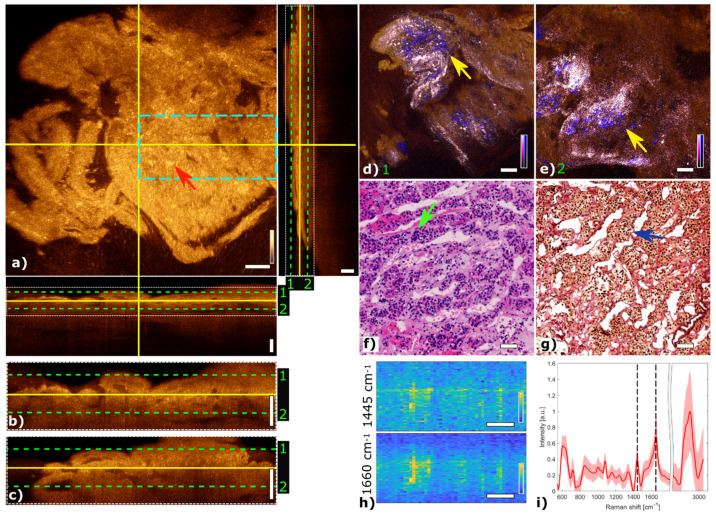
Multimodal investigation of pituitary gland. (**a**) OCT orthogonal view: en face image shows plane at the depth indicated in B-scans (depth profiles) with yellow lines. OCT en face and B-scans were averaged 5 times. Red arrow indicates a cell nest. The green dashed lines (1 and 2) in the OCT B-scans indicate the position of the overlaid OCT-MPM images. The blue dashed rectangle points out the region where the LSRM scan was performed. White dotted rectangles in OCT B-scans indicate positions of magnified regions of interest. (**b**,**c**) Magnified horizontal and vertical OCT B-scans. (**d**,**e**) MPM combining SHG and TPEF signals overlaid with OCT at different depths. NADH and FAD information (TPEF) reveals contrast corresponding to pituitary cells in blue, and collagen (SHG) shows the scaffold structure surrounding the cells in bright pink, respectively. Yellow arrows indicate cell nest with surrounding connective tissue. (**f**) Corresponding H&E-stained image of pituitary gland biopsy. Green arrow marks a cell nest. (**g**) Corresponding EVG-stained image giving contrast from connective tissue in pink. Blue arrow marks surrounding connective tissue. (**h**) LSRM images at 1445 cm^−1^ and 1660 cm^−1^ provide molecular contrast assigned to collagen, proteins and lipids, respectively. The corresponding Raman bands are shown and marked with black dashed lines on the mean Raman spectrum (**i**). All scale bars indicate 50 µm.

Figure 3 shows the multimodal imaging results of pituitary gland tissue and correlates the information to histology. The orthogonal OCT view shows a representative OCT en face slide at a depth of about 60 µm and the corresponding B-scans. Bright areas in OCT (Figure 3a) indicate changes in refractive indices which originate from cell nests or connective tissue, marked with a red arrow. The typical agglomerations of cells within the gland are clearly visible in the TPEF channel and appear as small blue dots in Figure 3d,e. The supporting matrix of connective tissue appears as pink in the SHG channel. The fused multimodal image in Figure 3d is acquired at a depth of approximately 30 µm and allows for precise identification of the different contrast mechanisms from each modality: cell groups with their surrounding connective tissue are clearly resolved in the MPM overlay and correlated with the OCT image (see yellow arrow). The features are also present in the H&E- and EVG-stained slices in Figure 3f,g, respectively (see green and blue arrows). The fused multimodal image in Figure 3e is measured at a depth of approximately 90 µm and reveals distinct depth-resolved changes: the nesting behavior and the gland meshwork in depth. LSRM images, see Figure 3h, are visualized at most notable discriminative Raman bands of interest as determined by the PCA (Figure 3i). The Raman band at 1660 cm^−1^ is known to be the vibration for Amide I, which can be linked to protein and collagen content. As cells exhibit collagen in the extracellular matrix, collagen is detected by LSRM, as well as the lipids and proteins indicated by the Raman bands at 1445 cm^−1^ and 1660 cm^−1^ [44]. The pattern of LSRM images indicates cell nests.

### 3.2. Pituitary Adenomas

In the following, two representative pituitary adenomas from two different subtypes are presented and discussed in detail.

#### 3.2.1. Mammosomatotroph Adenoma

**Figure 4:**
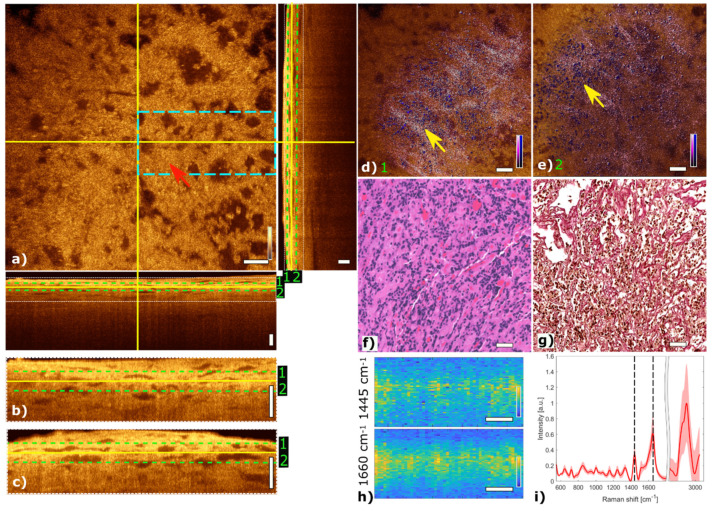
Multimodal investigation of a representative mammosomatotroph adenoma. (**a**) OCT orthogonal view: en face image shows plane at the depth indicated in B-scans (depth profiles) with yellow lines. OCT en face and B-scans were averaged 5 times. Red arrow indicates an area where single cells are visible. The green dashed lines (1 and 2) in the OCT B-scans indicate the position of the overlaid OCT-MPM images. The blue dashed rectangle indicates the region where the LSRM scan was performed. White dotted rectangles in OCT B-scans indicate positions of magnified regions of interest. (**b**,**c**) Magnified horizontal and vertical OCT B-scans. (**d**,**e**) MPM combining SHG and TPEF signals overlaid with OCT at different depths. NADH and FAD information (TPEF) reveals contrast corresponding to cells in blue, and SHG provides contrast from the surrounding tissue containing collagen in bright pink, respectively. Yellow arrows indicate single cells and disordered surrounding connective tissue. (**f**) Corresponding H&E-stained image of mammosomatotroph adenoma showing sparse granulation of cells. (**g**) Corresponding EVG-stained image with contrast from connective tissue in pink. (**h**) LSRM images at 1445 cm^−1^ and 1660 cm^−1^ provide molecular contrast assigned to collagen, proteins and lipids, respectively. The corresponding Raman bands are shown and marked with black dashed lines on the mean Raman spectrum (**i**). All scale bars indicate 50 µm.

Figure 4 shows multimodal imaging results of a mammosomatotroph adenoma and correlates the information to histology. The orthogonal OCT view shows a representative OCT en face slide at a depth of about 45 µm and the corresponding B-scans. The red arrow in the OCT image in Figure 4a marks a region where single cells are visible. The mammosomatotroph adenoma shows no cell nests in the TPEF channel. The homogenous cell pattern is visualized in blue. Collagen structures which are present in the SHG channel and color-coded in pink show a homogeneous pattern. The fused multimodal image in Figure 4d is acquired at a depth of approximately 30 µm and shows a homogeneous distribution of single cells across the sample which can also be seen in the OCT image (see yellow arrow) as small bright spots and is in good agreement with the H&E and EVG stained images in Figure 4f,g, respectively. The fused multimodal image in Figure 4e is detected at a depth of approximately 60 µm and shows single cells without a pronounced scaffolding structure as it is found in pituitary gland tissue, marked with a yellow arrow. The MPM as well as the H&E-stained images show the sparse granulated mammosomatotroph adenoma. LSRM images in Figure 4h show a homogenous spatial distribution.

#### 3.2.2. Gonadotroph Adenoma

**Figure 5:**
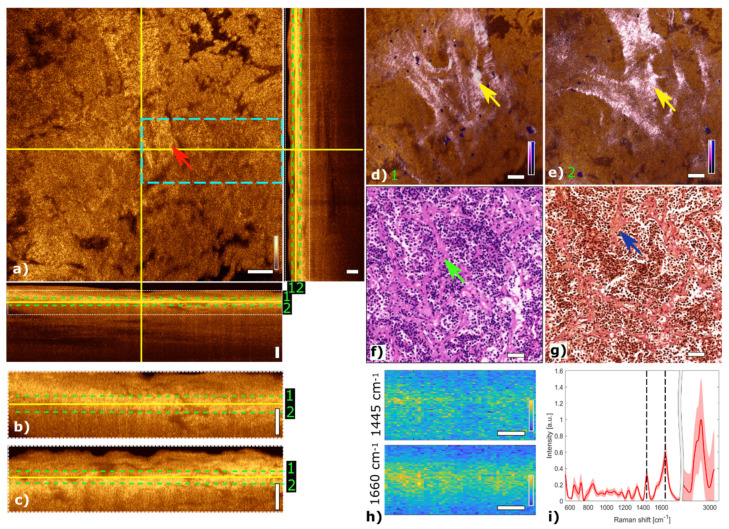
Multimodal investigation of a representative gonadotroph adenoma. (**a**) OCT orthogonal view: en face image shows the plane at the depth indicated in B-scans (depth profiles) with yellow lines. OCT en face and B-scans were averaged 5 times. Red arrow indicates connective tissue. The green dashed lines (1 and 2) in the OCT B-scans indicate the position of the overlaid OCT-MPM images. The blue dashed rectangle points out the region where the LSRM scan was performed. White dotted rectangles in OCT B-scans indicate positions of magnified regions of interest. (**b**,**c**) Magnified horizontal and vertical OCT B-scans. (**d**,**e**) MPM combining SHG and TPEF signals overlaid with OCT at different depths (1 and 2). NADH and FAD information (TPEF) reveals contrast corresponding to cells in blue, while collagen (SHG) shows the connective tissue in bright pink, respectively. Yellow arrows mark elongated structures which are connective tissue. (**f**) Corresponding H&E-stained image of gonadotroph adenoma. Green arrow indicates connective tissue which can also be seen in the corresponding EVG-stained image in (**g**) (see blue arrow). (**h**) LSRM images at 1445 cm^−1^ and 1660 cm^−1^ provide molecular contrast assigned to collagen, proteins and lipids, respectively. The corresponding Raman bands are shown and marked with a black dashed line on the mean Raman spectrum (**i**). All scale bars indicate 50 µm.

Figure 5 shows the multimodal imaging results of a gonadotroph adenoma and correlate the information to histology. The orthogonal OCT view shows a representative OCT slide at a depth of about 45 µm and the corresponding B-scans. The papillary structure can be identified by brighter regions marking papillary formations as indicated with a red arrow in the OCT image (Figure 5a). Due to changes in the refractive index within the tissue, the papillary structure of collagen becomes visible in the OCT image. MPM contrast in the fused multimodal images reveals distinct regions with high collagen content via SHG color-coded in pink, see Figure 5d,e. The papillary structure of connective tissue can be observed at different depths. The loss of cell nests is also observed in this adenoma in the TPEF channel. The fused multimodal image in Figure 5d is acquired at a depth of approximately 30 µm and shows pronounced elongated papillary structures, which are also present in the OCT image (yellow arrows) and H&E- and EVG-stained images in Figure 5f,g, respectively (green and blue arrows). The fused multimodal image in Figure 5e is detected at a depth of approximately 60 µm and also shows strong collagen signals indicated with the yellow arrow. LSRM images in Figure 5h show a reduced homogenous signal level.

### 3.3. Radiomic Analysis

To accomplish binary classification between healthy gland tissue and adenoma, a radiomic feature extraction was performed on a dataset of 174 2D planes. 149 of them originated from 25 different adenoma biopsies and the other 25 originated from 5 healthy pituitary gland biopsies. In each 2D plane 4 co-registered multimodal images were included: an SHG image, a TPEF image (meaning FAD and NADH fluorescent channels overlaid), a full MPM image (meaning SHG and TPEF overlaid) and an OCT en face image. 

**Figure 6:**
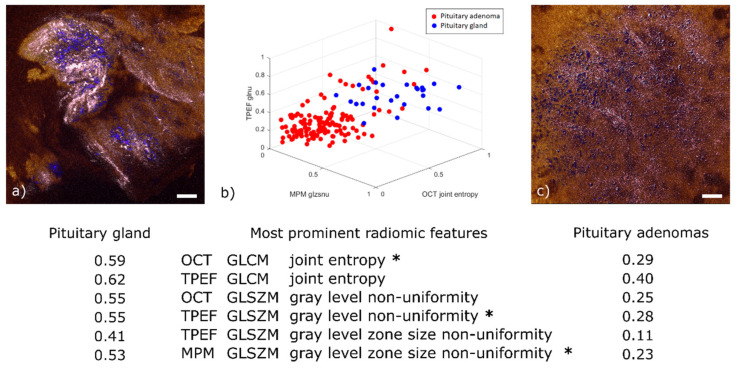
Radiomic analysis and comparison of representative pituitary gland and pituitary adenoma images. (**a**) OCT-MPM overlay of pituitary gland showing prominent cell nests (colored in blue) with surrounding connective tissue (colored in pink). (**b**) 3D scatter plot of three out of the six most prominent radiomic features. The selected features are marked with an asterisk in the table below. The table displays the average value for each of the six prominent features in pituitary gland and pituitary adenomas. All values normalized by their respective maximum. (**c**) Representative OCT-MPM overlay of pituitary adenoma (mammosomatotroph) displaying the loss of structure in connective tissue (color-coded in pink) and distribution of cells (color-coded in blue). Scale bars indicate 50 µm.

Among the 98 relevant radiomic features extracted from this dataset (*p*-values < 0.05), we determined 6 to be the most prominent (see table below Figure 6). For illustration purposes, Figure 6b displays a 3D scatter plot of three out of these six features. For ease of display, the values given for these features have been normalized to their own maximum. Two of them are the joint entropy of the gray level co-occurrence matrix (GLCM), which is a widely used heterogeneity measure in between neighboring pixels [45]. It was determined to be a prominent feature in both the TPEF and OCT images. The reason for this correlation is that OCT is sensitive to the local tissue density and TPEF is a measure of fluorescence intensity; regions with higher cell density also emit more fluorescence, everything else being equal. The normalized average values of this feature are higher for healthy tissue (0.59 in OCT and 0.62 in TPEF) than for adenoma (respectively 0.29 and 0.40). This behavior can be explained by a higher homogeneity in local tissue structure for adenoma samples (see Figure 6c).

The third and fourth significant features are the gray level zone size non-uniformity from the gray level size zone matrix (GLSZM). This feature implies a heterogeneity pattern not only across neighboring pixels—as in the case of GLCM—but also across the distribution of homogeneous sub-regions. This emerges from the nest structures formed by connective tissue around healthy cells (Figure 6a). It was found to be prominent in both TPEF and MPM images. This correlation was expected given that MPM is obtained by adding the SHG and TPEF channels. The normalized average values are higher in healthy tissue (0.41 in TPEF and 0.53 in MPM) than in pituitary adenomas (0.11 in TPEF and 0.23 in MPM). This finding is explained by the loss of nest structures observed for pituitary adenomas where similar occurrences of zones sizes imply a more uniform long-range pattern (see Figure 6c).

Finally, the gray level non-uniformity of the GLSZM, which is another measure of long-range organization, was also determined to be prominent in both TPEF and OCT. Once again, the normalized average values are higher for healthy tissue (0.55 in TPEF and 0.55 in OCT) than for adenoma (0.28 in TPEF and 0.25 in OCT). This is similarly explained by the loss of nest structures in adenomas.

### 3.4. Metabolic Status Based on NADH and FAD Composition

The metabolic status of a cell can be described using the relative concentration of the two molecules NADH and FAD [46,47]. The ratio between NADH and FAD is known to be an indicator for oncogenesis as cells divide faster and in an uncontrolled manner [29,30]. In our study, we calculated the TPEF intensity ratio between NADH and FAD. We corrected this ratio for relevant external factors that are average laser power, acquisition gain and detector spectral response to be able to compare the images acquired from the two acquisition channels. This calculation yielded the following results: 

$\frac{\mathrm{NADH}}{\mathrm{NADH}+\mathrm{FAD}}$ = 0.40 ± 0.11 for gland (*n* = 5), 0.74 ± 0.08 for lactotroph adenomas (*n* = 5), 0.85 ± 0.08 for null cell adenomas (*n* = 5), 0.57 ± 0.05 for gonadotroph adenomas (*n* = 5), 0.77 ± 0.09 for somatotroph and mammosomatotroph adenomas (*n* = 5), and 0.86 ± 0.10 for corticotroph adenomas (*n* = 5). The results were all significant with *p*-values < 0.05.

### 3.5. Raman Spectral Analysis

**Figure 7:**
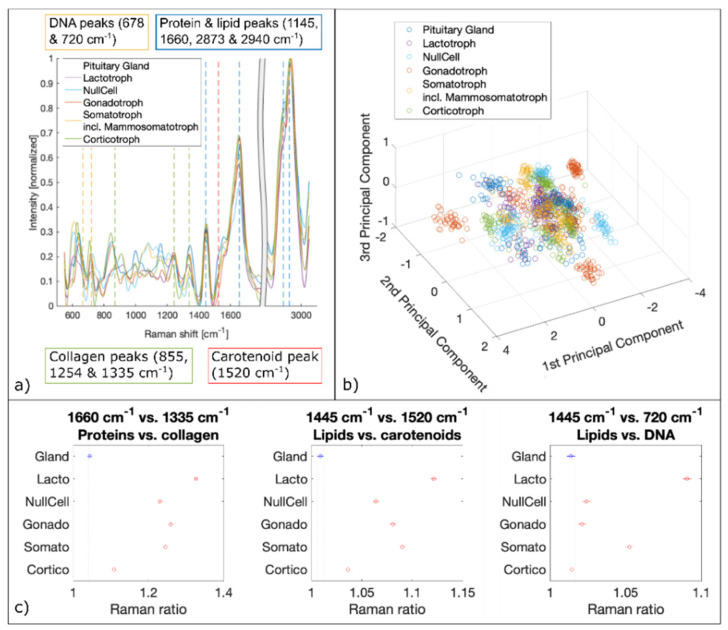
Spectral analysis of Raman data. (**a**) Mean Raman spectra of pituitary gland and pituitary adenoma subtypes. Raman bands of DNA, collagen, carotenoid, proteins and lipids are marked which are identified by PCA as significant differences. (**b**) 3D scatter plot of the three first principal components derived from PCA. (**c**) Raman ratios identified as the most significant discriminators via PCA.

We analyzed about 2100 spectra per biopsy. Therefore, a total of about 10,000 Raman spectra per pituitary gland and adenoma subtypes were fed to the PCA followed by SVM. The spectral analysis of LSRM shows distinct differences between spectral profiles and is presented in Figure 6. Based on PCA, see scatter plot in Figure 7b, important peaks for discrimination between pituitary gland and pituitary adenoma subtypes were identified: the peaks assigned to DNA (678 cm^−1^ and 720 cm^−1^), collagen (855 cm^−1^, 1254 cm^−1^ and 1335 cm^−1^), carotenoid (1520 cm^−1^), protein and lipids (1445 cm^−1^, 1660 cm^−1^, 2873 cm^−1^ and 2940 cm^−1^) are significant. Table 2 gives an overview of all detected Raman bands and the corresponding vibrational modes.

To confirm the significance of these peaks for differentiation between pituitary gland and adenomas and the prediction capability for different adenoma subtypes, ratios based on the most discriminating Raman bands were calculated and plotted [48,49,50,51]. Significant Raman ratios (*p*-values < 0.05) included 1660 cm^−1^ versus 1335 cm^−1^, 1445 cm^−1^ versus 1520 cm^−1^ and 1445 cm^−1^ versus 720 cm^−1^, see Figure 7c. The Raman ratio 1445 cm^−1^ versus 1520 cm^−1^ is assigned to the difference between lipids and carotenoids, respectively. Lipids and carotenoids have been found to be an indication of carcinogenesis [52,53]. Our findings show higher levels of lipids in pituitary adenomas compared to pituitary gland. To confirm higher lipid levels in adenomas and to test for DNA content detected with LSRM, Raman ratios 1445 cm^−1^ versus 720 cm^−1^ were calculated. 720 cm^−1^ is a vibrational mode which is assigned to DNA [54,55]. Our results show elevated lipid levels for four out of five pituitary adenoma subtypes. Only corticotroph adenomas did not follow this trend showing no significant difference in respect to pituitary gland. The Raman ratio 1660 cm^−1^ versus 1335 cm^−1^ indicates the ratio between protein and collagen composition which was found to be significantly increased for all pituitary adenoma subtypes compared to pituitary gland.

### 3.6. Multiparametric Biomarker

**Figure 8:**
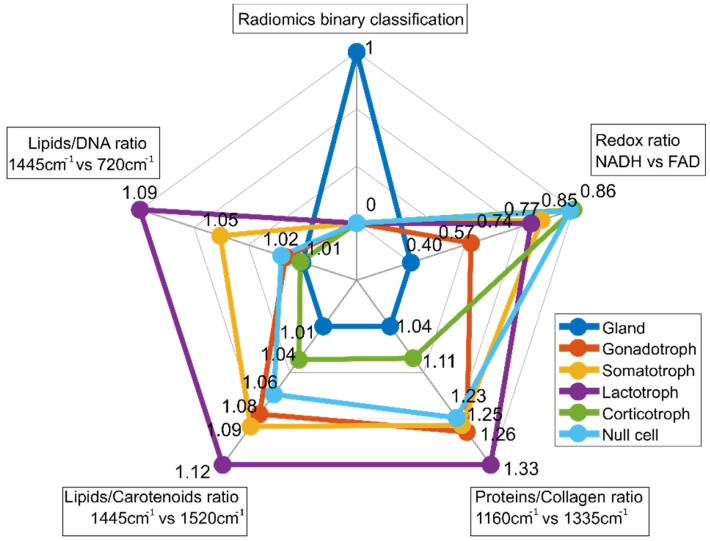
Spider plot for differentiation between pituitary gland and pituitary adenoma subtypes. It combines binary classification by radiomic analysis, TPEF signal analysis and RS. MPM contrast arises from SHG and TPEF signals, revealing information about amount of collagen and collagen distribution as well from differences in the NADH and FAD signals (TPEF NADH vs. FAD), respectively. For subclassification into pituitary adenoma subtypes, Raman ratios including collagen, protein, lipid, carotenoid and DNA ratios were identified as relevant.

For multiparametric biomarker evaluation, the results are represented in a spider plot which gives an idea of the fingerprint of each tissue type. The first entry of the spider plot of Figure 8 ‘Radiomics binary classification’ represents the binary classification results between pituitary gland and pituitary adenomas resulting from radiomic feature extraction. The second entry (‘Redox ratio NADH vs. FAD’) shows a lower value (0.40) for pituitary gland, while for all pituitary subtypes, the value is increased and varies between 0.57 and 0.86. Corticotroph adenomas show the highest value. The relationship between NADH and FAD gives an idea as to the metabolic state of cells and is calculated from TPEF measurements. Raman ratios that were determined to be significant are included in the spider plot as well. Spider plot entries 3 (‘Proteins/Collagen ratio’), 4 (‘Lipids/Carotenoids ratio’) and 5 (‘Lipids/DNA ratio’) visualize differences in protein, collagen, lipid, carotenoid and DNA, respectively. In general, Raman ratios for pituitary gland yield lower ratios compared to pituitary adenomas. Based on these findings our multiparametric biochemical biomarker is established combining all modalities. The discriminatory markers between pituitary gland and pituitary adenoma subtypes, and also within the subtypes, are clearly visible.

### 3.7. Classifier Performance

First-level classification was performed via radiomic analysis and feature extraction to discriminate between pituitary gland and adenomas in real time. Table 3 provides an overview of classification parameters (accuracy, sensitivity and specificity). Upon binary classification between pituitary gland and adenomas, second-level classification by a PCA-SVM on LSRM data was used to discriminate pituitary adenoma subtypes with accuracies ranging between 97–99%. The high accuracy values could be explained by the small size of the dataset: the pituitary gland was represented with five distinct datasets from five glands and five datasets from five adenomas per subtype were fed to the classifier. Reduced areas of OCT-MPM overlays were scanned for this high-performance multi-class classification to find a compromise between completely screening the entire biopsy and reasonable acquisition times of up to 10 min.

## 4. Discussion

Our integrated multimodal OCT, MPM and LSRM platform was used to characterize pituitary gland and pituitary adenomas in a fast and label-free manner. OCT is a powerful optical imaging modality performing cross-sectional imaging of tissue morphology on a micrometer scale and cm^2^ FOV in real-time with penetration depths up to 1–2 mm. It is an interferometric technique with contrast arising from refractive index changes within the tissue. We used OCT for fast 3D navigation across the specimen. The live preview allowed for identification of relevant morphological and textural features arising from the tissue structure. This modality was used as a first discriminator between healthy and tumorous tissue. In particular, the presence or absence of collagen nest structures was found to be the main discriminator between gland and adenoma. Indeed, the pituitary gland is characterized by cells gathered into glandular nests surrounded by the extracellular matrix containing collagen [24,56]. Recently, it has become possible to discriminate the pituitary gland from pituitary adenomas with an accuracy of at least 80% with OCT, critically depending on resolution [19]. However, the cited article produced neither a sensitivity nor a specificity value; this means the independent predictive ability to classify glands vs. adenomas is unknown. In our study, the OCT en face images are fed into our radiomic analysis along with three other co-registered modalities to achieve higher specificity.

MPM is another method of choice for highly contrasted micrometer investigation of intact tissue. It combines the technique of laser scanning microscopy with long wavelength excitation to provide improved depth penetration and reduced photodamage compared to confocal laser scanning microscopy. In our study, we added TPEF and SHG microscopy as commonly applied MPM methods to OCT to provide additional contrast. We recorded 2D MPM planes at different depths within the OCT volumes. TPEF occurs when two photons with a sum energy satisfying the transition energy required to promote a fluorophore from the ground to the excited state interact simultaneously with a given fluorescent molecule. In our study, we investigated the endogenous molecules NADH and FAD that are inherently expressed in tissue. The two mitochondrial metabolic coenzymes are autofluorescent and important optical biomarkers for estimating the redox state of a cell. In this way, predictions about the metabolic state can be made. We observed direct correlation of the autofluorescence signatures between pituitary gland and adenomas under the excitation wavelengths of 760 nm and 865 nm with two emission channels centered at 445 nm and 550 nm, respectively. The results revealed a significant increase in the optical redox ratio from gland to adenoma. Additionally, significant differences between the adenoma subtypes were observed. In corticotroph adenomas the ratio yielded the highest value, while null cell adenomas showed only a moderate elevated value. The fact that this ratio is lower in healthy tissue is in good agreement with the literature, as adenoma tissue is expected to have a higher metabolic state as cells are proliferating fast [29,30]. Differences between gland and adenomas are already evident from the images. The distribution pattern of cells is more homogenous in adenomas, showing no cell nest, as in the gland. Additionally, in the histopathological analysis, tumor cells show diffuse patterns. Mammosomatotroph adenomas are a subtype of somatotroph adenomas and are characterized by the loss of cell nests and change in collagen content and distribution. Somatotroph adenomas show sparse or dense granulation [24,56]. This behavior is not only observed in the OCT images, resulting in a more homogenous pattern compared to pituitary gland, as recently reported [19], but also in the TPEF images. Another MPM technique is based on SHG: two photons instantaneously transfer their energy to a single photon of half the wavelength by interacting with a non-centrosymmetric object. Unlike fluorescence, SHG is not an absorptive and emissive process but requires highly ordered molecular structures with particular symmetry. Collagen is the most common biological structure fulfilling this requirement. Hence, in our multimodal approach we gain deeper insight into the tissue composition and alterations. Indeed, morphological features revealed via OCT could be assigned to changes in reticular fibers composed of type III collagen detected by SHG. The structure and textural features of collagen are known to change in the pituitary gland upon oncogenesis to a pituitary adenoma [24]. In our representative fused multimodal images we see clear differences in the collagen distribution and changes in the extracellular matrix where collagen is the most abundant fibrous protein [57]. Pituitary adenomas show distensible meshes in reticular framework and incomplete reticular fibers compared to pituitary gland [17]. The growth pattern could be diffuse, sinusoidal, papillary, adenoid or nesting. In addition, gonadotroph adenomas are known for prominent elongated structures in papillary or pseudo-rosette patterns [24,58]. During oncogenesis, connective tissue (e.g., collagen) is ripped apart, explaining this behavior [24,56]. Radiomic analysis of fused OCT, TPEF and SHG data showed in a more objective way the capability of the combination of these imaging modalities to sense these subtle changes by performing binary discrimination between pituitary gland and adenomas with 83% specificity, 93% sensitivity and 88% accuracy.

Upon first-level binary classification provided by radiomic analysis based on OCT and MPM images, LSRM provided additional contrast about the molecular composition of tissue for multi-class classification. This technique gives a wealth of highly specific information but has certain limitations. Indeed, since Raman scattering is characterized by very small interaction cross sections, this technique intrinsically requires long acquisition times. With our LSRM approach we could increase the acquisition speed by a factor of ten. Furthermore, we performed LSRM on a reduced FOV to limit acquisition times to a maximum of ten minutes. These ROIs were used for further discrimination of pituitary adenoma subtypes. PCA followed by SVM allowed for classification of pituitary gland and pituitary adenoma subtypes, namely lactotroph, null cell, gonadotroph, somatotroph and mammosomatotroph and corticotroph adenomas and identified relevant Raman bands. Raman ratios were calculated for these Raman bands including collagen, protein, lipid, carotenoid and DNA. These results show that LSRM reflects the presence and degree of tissue pathology. It might facilitate the understanding and identification of subtle changes taking place in pituitary adenoma subtypes. Our results show elevated lipid levels for all pituitary adenoma subtypes. Also changes in the collagen content based on Raman spectral analysis are in agreement with previous studies [20]. Second-level classification by PCA-SVM showed the high potential of the proposed approach to identify the different subtypes of pituitary adenomas. In a previous study, comparable accuracies ranging from 84–99% were achieved [20]. Therefore, our results are in good agreement with the previous study on pituitary gland and adenoma subtypes. The loss of cell nests in pituitary adenomas can also be observed in the LSRM images. The pattern in LSRM images of mammosomatotroph and gonadotroph adenomas at the 1445 cm^−1^ and 1660 cm^−1^ Raman bands show a more homogeneous signal distribution compared to pituitary gland LSRM images. They provide information about the spatial distribution of lipids and proteins. While for the pituitary gland and the mammosomatotroph adenoma the Raman signal at 1445 cm^−1^ assigned to lipid molecules provides clearly visible contrast, for the gonadotroph adenoma the signal levels are reduced.

In our study, we visualized our established multimodal biomarker in a spider plot to provide a comprehensive picture of the morphological, metabolic and molecular information that could be obtained from our imaging platform. The plot clearly indicates the differences and classification capabilities between pituitary gland and pituitary adenoma subtypes by means of five distinct entries. The ratio between NADH and FAD is an important biomarker in the discrimination between pituitary gland and adenomas. Our multimodal optical imaging approach offers ultrahigh-resolution images comparable to histopathology to fully assess the pituitary gland and pituitary adenoma subtypes. The single optical path in our platform with a single high NA microscope objective facilitates co-registration and by that co-localization of the single modalities. Color-coded MPM images to mimic conventional H&E staining can be a step towards “virtual histology”. The use of an advanced deep learning algorithm on large sample numbers could facilitate this approach.

Concerning other methods of structural analysis, magnetic resonance spectroscopy (MRS) was performed to evaluate the correlation with intraoperative findings. In the first quantitative study, Stadlbauer et al. found that due to the concentration of choline-containing compounds, this imaging technique could provide information on the proliferative potential of pituitary adenomas [59]. Further investigations found that heterogeneous signals in MRS analysis could possibly deliver information about the subtype of pituitary adenoma [60] and that the choline to creatine ratio may predict the rate of expression of SSTR2 and therefore give information about the effect of medical therapy [61]. However, this method is limited to a certain voxel size and therefore, smaller tumors deliver incorrect results due to the presence of the surrounding structures of the skull base.

Since the pituitary tissue is highly scattering, only a limited penetration depth of approximately 300 µm could be achieved with our platform. This was due to the combination of center wavelength and the use of a high NA objective that was used as a trade-off between the system requirements for the different modalities, ultrahigh resolution and sufficient penetration depth. This reduced penetration depth clearly poses a limitation to our multimodal investigation. Moving towards longer wavelengths, e.g., 1300 nm, should reduce scattering significantly and increase penetration depth to 1–2 mm. We decided for a center wavelength of 800 nm to achieve almost isotropic ~1 µm resolution to be comparable to histopathology. Current state-of-the-art light source technology would increase the resolution to 5 to 10 µm. Future investigation should explore longer wavelengths. Another limitation is the acquisition speed of RS. With our LSRM approach we could increase it but we are still limited in the FOV and prone to artifacts caused by movement during the acquisition. Furthermore, since MPM and RS collect photons with a photomultiplier or spectrometer, they are techniques that are highly sensitive to ambient light. Since the Raman effect is very weak, this limitation could pose a problem in the operation theatre especially for the RS when seeking in vivo applications. Maximum permissible exposure times also have to be carefully evaluated.

## 5. Conclusions

Our synergistic combination of advanced optical imaging techniques in a single platform to facilitate co-registration provides complementary morphological, metabolic and molecular contrast to analyze and distinguish pituitary gland from pituitary adenoma subtypes. While the data and analysis presented in this paper are preliminary and further investigation with a larger number of samples is required, the proposed multimodal approach based on OCT, MPM and LSRM has a high potential for diagnostics and classification of pituitary adenomas. This multimodal platform with integrated radiomic analysis could pave the way towards advanced real-time 3D intra-surgical pituitary tissue characterization. Given enough data, this tool could be used to build automatized radiomic-based predictive models in the future. As our approach could be performed intra-surgically in real-time, this pilot study aims to demonstrate the feasibility of our multimodal platform for pituitary imaging as a fast alternative compared to conventional histological procedures. Future works could target the miniaturization of the modalities aiming for their in vivo application in endoscopic approaches, thereby assisting surgeons in the operating theatre.

## Figures and Tables

**Table 1:** System parameters.

Modality	Excitation Wavelength	Detection Wavelength	Resolution	Contrast Mechanism
OCT	700–900 nm	700–900 nm	lateral: 1 µm axial: 2.2 µm	Changes in index of refraction (e.g., morphology)
SHG	865 nm	432 nm	lateral: 1 µm axial: 2.7 µm	Non-Centrosymmetric structure (e.g., collagen)
TPEF	760/865 nm	445/550 nm	lateral: 1 µm axial: 2.7 µm	Fluorescence of NADH/FAD
LSRM	785 nm	817–1050 nm	spectral: 0.5 nm lateral: 10 µm	Molecular

**Table 2:** Overview of Raman bands, corresponding assignment and vibrational modes of pituitary gland and pituitary adenomas [55].

Wavenumber [cm^−1^]	Assignment	Vibrational Mode
678	Ring breathing of DNA	
720	DNA	
855	Ring breathing of tyrosine, protein, carbohydrates, and collagen	ν(C-C)
937	Protein, collagen backbone	ν(C-C)
1004	Phenylalanine	ν(C-C), aromatic ring breathing
1093	Nucleic acids, phospholipids	ν(PO_2_^−^), ν(C-C), ν(C-N)
1160	Proteins, tyrosine	ν(C-C), ν(C-N)
1254	Protein, collagen	Amide III (mix of ν(C-N) and δ(N-H))
1335	Collagen, protein	τ(CH_3_/CH_2_), ω(CH_3_/CH_2_)
1445	Fatty acids, protein, lipid	δ(CH_2_/CH_3_)
1520	Carotenoids	ν(C-C)
1660	Unsaturated fatty acids, protein, lipids	Amide I (ν(C = C), ν(C = O))
2873	Lipids	ν(CH_2_)
2940	Proteins, lipids	ν(CH_3_)

**Table 3:** Performance of radiomics, and PCA-SVM on Raman spectra. Please note that radiomics performance values are from a 100-fold Monte Carlo cross-validation, while PCA-SVM performance values demonstrate the discrimination power of Raman spectra.

	Accuracy	Sensitivity	Specificity
Radiomics[binary]	88%	93%	83%
Raman[pituitary gland]	99%	96%	99%
Raman[lactotroph adenoma]	97%	95%	97%
Raman[null cell adenoma]	99%	99%	99%
Raman[(mammo)somatotroph adenoma]	99%	99%	99%
Raman[gonadotroph adenoma]	97%	91%	99%
Raman[corticotroph adenoma]	99%	95%	99%

## Data Availability

Data available upon reasonable request.
